# How Does Botulinum Toxin Inhibit Itch?

**DOI:** 10.3390/toxins14100701

**Published:** 2022-10-12

**Authors:** Parisa Gazerani

**Affiliations:** 1 Department of Life Sciences and Health, Faculty of Health Sciences, Oslo Metropolitan University, 0130 Oslo, Norway; parisaga@oslomet.no or gazerani@hst.aau.dk; 2 Department of Health Science and Technology, Faculty of Medicine, Aalborg University, 9220 Aalborg East, Denmark

**Keywords:** botulinum neurotoxin, itch, anti-pruritic, mechanism of action

## Abstract

Two decades after reports of the anti-pruritic effects of botulinum neurotoxins (BoNTs), there is still no approved product for the anti-itch indication of BoNTs, and most clinical case reports still focus on the off-label use of BoNTs for various itchy conditions. Few randomized clinical trials have been conducted with controversial results, and the beneficial effects of BoNTs against itch are mainly based on case studies and case series. These studies are valuable in presenting the potential application of BoNTs in chronic pruritic conditions, but due to the nature of these studies, they are categorized as providing lower levels of evidence or lower grades of recommendation. To obtain approval for the anti-pruritic indication of BoNTs, higher levels of evidence are required, which can be achieved through conducting large-scale and well-designed studies with proper control groups and established careful and reliable primary and secondary outcomes. In addition to clinical evidence, presenting the mechanism-based antipruritic action of BoNTs can potentially strengthen, accelerate, and facilitate the current efforts towards further investments in accelerating the field towards the potential approval of BoNTs for itchy conditions. This review, therefore, aimed to provide the state-of-the-art mechanisms underlying the anti-itch effect of BoNTs from basic studies that resemble various clinical conditions with itch as a hallmark. Evidence of the neuronal, glial, and immune modulatory actions of BoNTs in reducing the transmission of itch are presented, and future potential directions are outlined.

## 1. Introduction

### 1.1. Botulism, Clostridium Botulinum, and Botulinum Toxin

An illness characterized by muscle paralysis following the consumption of spoiled sausage was first termed botulism by Muller [1]. Botulus means lunch meat, salami, sausage, and similar in Latin [2], and it most likely dates back to earlier historical concerns about foodborne toxicity in the Byzantine era [3]. A comprehensive description of a somewhat similar illness by Justinus Kerner [1], is in line with what is currently considered in the diagnosis of botulism, which is marked by muscle weakness or paralysis, swallowing difficulty, and signs of autonomic dysfunction, such as dry mouth [4]. Krener was the first to propose that the illness is potentially caused by a biological toxin [5]. Emile Van Ermengem, in 1895, provided the first description of the organism that could cause botulism, an anaerobic Gram-positive, rod-shaped bacterium, which he named bacillus botulinum [6]. The name was changed in 1924 by Ida Bengstrom to *Clostridium botulinum*, based on the spindle-like shape of the bacteria [6], where kloster in Greek means spindle. The purification and development of botulinum toxin, the toxin from clostridium botulinum, was eventually dated back to the time of World War II, when it could potentially be used as a biological weapon [7,8]. Carl Lamanna and Richard Duff were the first to extract and crystalize botulinum toxin [9]. Later in 1946, Edward Shantz and Erik Johnson could purify larger amounts and also further refine the toxin for clinical research [10]. Burgen and his colleagues, in 1949, explained how the toxin can produce muscle paralysis and presented the effect of the toxin on the neuromuscular junction and blockade of acetylcholine (Ach) release [11]. In 1964, when Daniel Drachman demonstrated muscle weakness in the hind limb of a chicken following toxin administration, which was dose-dependent, Allen Scott and his colleagues became interested in using it for strabismus [7,12]. The first paper on this subject was published in 1980, presenting a clinical trial’s results of 67 patients, where a selected injection of BoNT into eye muscles could correct strabismus [12]. Evidence of the benefits of the BoNT injection, as shown in blepharospasm and hemifacial spasm, finally led to the approval of botulinum toxin-A by the FDA for conditions of strabismus, blepharospasm, and hemifacial spasm in 1989 [13,14]. Since then, toxin product development and testing for various medical conditions has followed, which has resulted in FDA approvals [15]. There are also various off-label uses of BoNT in various medical fields [16,17]. Along the way, efforts by basic scientists have resulted in presenting the molecular structures of botulinum toxins and their mechanisms of action for various clinical conditions [18,19,20].

Several toxin serotypes are produced by *Clostridium botulinum* that, from the immunological point of view, are considered distinct and are named A, B, C1, C2, D, E, F, and G [21]. All serotypes are neurotoxins, except for C2. Human botulism is known to be caused by serotypes A, B, E, and rarely F, while botulism in fish, birds, and non-human mammals is mainly caused by serotypes C1 and D [22]. Botulinum neurotoxin serotype A (BoNTA) is the most toxic substance known to man with an estimated intravenous lethal dose (LD50) of 1–2 nanogram per kilogram (ng/kg) in humans [23]. Now, however, the lethal toxin has been developed as a safe medicinal product for clinical pharmacotherapy in a large number of medical conditions in humans [24,25], and it is also used in the field of veterinary medicine [26].

### 1.2. Botulinum Toxin Products and Uses

Several products are marketed around the globe with different product characteristics [27,28,29,30]. The chronological footprint tracking of the US-marketed botulinum neurotoxins approved by the FDA [31,32] shows OnabotulinumtoxinA (Botox) by Allergan Inc., approved for several conditions such as blepharospasm, hemifacial spasm, strabismus, cervical dystonia, glabellar lines, axillary hyperhidrosis, chronic migraine, upper limb spasticity, neurogenic bladder, lateral canthal lines, overactive bladder, lower limb spasticity in adults, forehead lines, and upper limb spasticity in children. IncobotulinumtoxinA (Xeomin) by Merz Pharma was approved by the FDA for a number of conditions such as cervical dystonia, blepharospasm, glabellar lines, upper limb spasticity in adults, and sialorrhea. Other approved products are AbobotulinumtoxinA (Dysport) by Ipsen Biopharmaceuticals, RimabotulinumtoxinB (Myobloc/Neurobloc) by US World Med-Solstice, and PrabotulinumtoxinA (Jeuveau) by Evolus Inc.

There are several conditions for which BoNT is not yet approved for clinical use, but off-label use has been presented, with beneficial effects, for example, in dermatology [33,34,35], primary headaches other than migraine [36], depression [37], and neuropathic pain [38,39]. One large area in which the off-label use of BoNT has been practiced for the last two decades is dermatology, for various conditions that appear with or without itch [40,41,42,43]. This focused review is dedicated to conditions accompanied by itch. Please consider that the use of BoNT in the field of cosmetology [44] is also active, but it is not the focus of this review. Interested readers are referred to a recent publication in this domain [45]. In the following, clinical evidence from the literature is presented first to indicate the current status for the use of BoNT in clinical conditions with itch. Thereafter, evidence is presented from basic science to outline the mechanistic basis for the antipruritic effects of BoNT. The purpose of this review is to accelerate the work in the field and motivate progression towards presenting the mechanism-based antipruritic action of BoNTs, which can potentially strengthen, accelerate, and facilitate their approval for itchy conditions.

## 2. Clinical Evidence for the Use of BoNT in Clinical Conditions with Itch

Itch is a sensory modality that is also called pruritus [46]. It often appears as an uncomfortable and unpleasant sensation and usually provokes a strong desire to scratch [46,47]. Dermatological conditions are often accompanied by itching [48]. Chronic itch [49], in particular, reduces the overall quality of life in affected patients, such as patients with atopic dermatitis (AD) [50,51]. Several types of itch have been defined, e.g., pruriceptive itch, neurogenic itch, neuropathic itch, and psychogenic itch [52,53]. This classification has been mainly based on the underlying mechanisms together with clinical manifestations [53]. Itch accompanies some systemic disorders, for example, some chronic kidney and liver diseases [54]. Diabetic neuropathy and shingles are among neurological disorders with itching as one of their debilitating symptoms [55,56,57]. Although probably not fully accurate, the first report of the antipruritic effect of Botulinum Toxin Type A (BoNTA) appeared in 2002, which presented the beneficial effects of BoNTA for lichen simplex in an open-label pilot study [58]. In the same year, the antipruritic effect of BoNTA was reported in hand dyshidrotic dermatitis [59]. Following these initial reports of the antipruritic effect of BoNTA, further investigations have continued [43,60], and successful treatments are being reported in the literature for many other dermatological conditions, such as Hailey–Hailey disease [61] and psoriasis [62].

A limited number of review articles are available, for example, the 2017 review on botulinum toxin off-label use in dermatology [63]. Another review [64] from the same year is also available, with a focus on novel indications of BoNT in dermatology. The most recent systematic review came out in 2021 [60]. This review included 167 studies from 1994 to 2020, and based on the evidence, diseases for which BoNTs could have therapeutic potentials were listed. These also included conditions with itch (e.g., post-herpetic neuralgia, notalgia paresthetica, Hailey–Hailey disease, and psoriasis) [60]. Most of these reports are based on case studies or case series that are considered to have an evidence level of three or four; hence, the grade of recommendation for use will be C and D [65]. On the other hand, there are cases of randomized control trials where results show no statistically significant effect of BoNT on itch, for example, the level of itch on the visual analog scale (VAS) or hyperpigmentation in notalgia parasthetica [66]. It is not clear as to what reason similar results of observational or case series are not repeated when studies are conducted as RCTs. This might be related to a true lack of effect, or other influential factors such as criteria for inclusion of participants (diversity, responsiveness, or lack of response) or selection of outcome measures (sensitivity, specificity) that might mask a statistically significant antipruritic effect. A review from 2018 [67], with a focus on localized chronic itch, evaluated available studies on the effect of BoNTA following intradermal administration. The authors found 25 studies conducted between 1996 and 2016 [67], where 11 articles were identified as relevant for further evaluation. This review [67] followed PRISMA and rated the studies based on the grade of recommendation. The toolkit provided by the Oxford Centre for Evidence-based Medicine Levels of Evidence was used in this review [67] to grade each study. The findings [67] showed that prospective observational studies and case reports mostly resulted in complete relief, strong improvement, and long-lasting effects (Table 1).

Collectively, systematic and non-systematic literature reviews, including all types of studies (e.g., case reports, case series, and RCTs), show that many applications of BoNT for dermatological conditions with and without itch are still off-label. Moreover, it seems that in the majority of cases, BoNTA might not be considered first-line therapy, but perhaps an option for patients with persistent or recurrent issues that remain unsolved or irresponsive to other treatments [43,76]. Toyama et al., in a recent review [76], presented a long list of available options for troublesome itch, where, among other options (e.g., medications used for depression and phosphodiesterase-4 inhibitors), BoNTA is also listed [76]. These authors also indicated that itch was relieved by BoNTA in patients with several conditions, such as chronic lichen simplex, inverse psoriasis, and post-burn itch [40,58,71]. As outcome measures, when the eczematic area and severity index (EASI) was used to determine the therapeutic effect, BoNTA reduced this score in chronic lichen simplex. Moreover, in inverse psoriasis, BoNTA reduced the PASI (psoriasis area and severity index) [40]. Pruritus and hyperhidrosis were both shown to be diminished in pediatric Fox–Fordyce disease [77]. BoNTA could also reduce itching related to keloid scars [78], in particular, when it was combined with triamcinolone [79], against atopic dermatitis [80] and post-burn itch [68].

BoNTs provide long-term therapeutic effects, and this characteristic is in favor of patient compliance [81]. However, it can be a costly treatment, and its comparable effects with other available agents might place BoNTs in the third or fourth line of therapy. It is too early to recommend the regular use of BoNTs at this stage, but it is not unlikely that we will witness the appearance of more reports on conditions in which stronger evidence exists for the beneficial effects of BoNTs. Further clinical trials must consider careful design and patient inclusion criteria, safe and effective dosing, and an optimal interval in repeated administration. In addition, a strategy for determining and implementing primary and secondary outcome measures would help in the more accurate identification of responders. The determination of influential factors, such as age, gender, and ethnic background, would also aid in targeting the right group of patients for the optimal effect. The formation of neutralizing antibodies [82] and the influence on safety and efficacy must also be taken into consideration for future clinical studies.

## 3. Mechanism(s) of Action of Botulinum Neurotoxins

As summarized above, BoNTA has been shown to reduce itch in several clinical dermatological conditions with itch as a common troublesome symptom [67,76]. Therefore, the next logical question would be to ask how this occurs [43,83]. Before addressing this question, two main clarifications are needed: (1) how botulinum toxin interacts with cells, receptors, and neurotransmitter release, and (2) how itch is generated and transmitted. 

In the following section, the mechanism of action of BoNT in inhibiting the Ach release at the neuromuscular junction [84] is presented first. This is the proposed mechanism underlying muscle paralysis that can lead to respiratory failure and death in botulism [4,85]. The muscle relaxation effect of BoNT explains its medical use for several conditions, such as spasticity and strabismus, where abnormal or excessive muscle spasm is problematic [86,87]. Secondly, to understand the inhibitory action of BoNTA on itch, itch mechanisms are presented. 

### 3.1. Mechanism of Action of Botulinum Neurotoxin in Blocking Neurotransmitter Release

**Figure 1:**
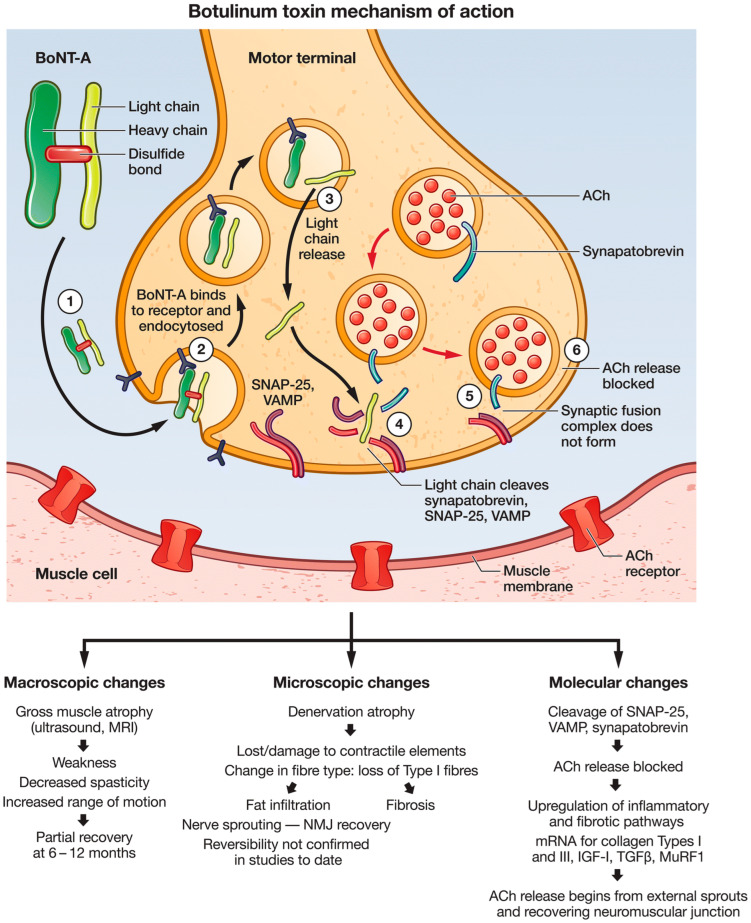
Botulinum toxin type A (BoNT-A) mechanism of action. The BoNT-A heavy chain is shown in green and the light chain in yellow, linked by a disulfide bond. Acetylcholine (Ach), the neurotransmitter that is blocked by BoNT-A, is shown as red dots within a circular vesicle in the nerve terminal. The effects of chemodenervation via injection of BoNT-A are summarized at macroscopic, microscopic and molecular levels. SNAP 25, soluble N-ethylmaleimide fusion protein/attachment protein; VAMP, vesicle-associated membrane protein. Reused from [87] for non-commercial/educational purposes under a Creative Commons license (Attribution-Noncommercial), Springer Nature.

Botulinum toxin consists of two chains, light and heavy chains, that are connected through a disulfide bond. The heavy chain, from its C-terminal region, binds to receptors at the presynaptic neurons [88]. A two-step receptor binding has been proposed: the first is binding to polysialoganglioside (PSG), which is followed by binding to SV2 (synaptic vesicle glycoprotein) [88]. This binding forms a complex that undergoes an endocytosis process. Within the cytoplasm, the disulfide bond is cleaved, permitting the light chain to act on the SNAP-25 (synaptosomal-associated protein-25) and cleave it. This is one of the protein components required for the vesicle fusion and exocytosis process. Interestingly, different serotypes cleave different proteins, which are Soluble N-ethylmaleimide-sensitive factor Attachment protein REceptors (SNAREs) [89]. Once vesicle fusion is prohibited, there is no vesicular release of transmitters from nerve terminals [18,19,90,91]. Figure 1 presents the proposed mechanism of action of BoNT in blocking the Ach release [87].

Identifying the blockade of Ach by BoNTA has sparked the interest of some researchers to look into other potential neurotransmitters that are blocked by BoNT. In one of the active areas, pain research, the analgesic action of BoNT was investigated extensively [92]. BoNTA shows analgesic efficacy in various pain conditions, such as musculoskeletal pain [93], dental medicine [94], and neuropathic pain [95], but it is only FDA approved for chronic migraine [96,97], among other headache and related disorders [98]. The proposed mechanism of action of BoNTA for migraine [99,100,101] has also been postulated by several researchers based on findings from basic and clinical research [102,103,104]. At a cellular–molecular level, some of the identified neurotransmitters and pathways targeted by BoNTA in pain are applicable in explaining the antipruritic effects of BoNTA [43]. This is perhaps because itch and pain interact and share some similarities while holding to the uniqueness of each modality [105,106,107,108]. Our studies from cells to animals and human experimental models of pain [83,109,110,111,112,113,114] revealed that BoNTA inhibits the release of several neurotransmitters involved in pain, including glutamate [113,114]. Other studies have also shown that BoNTA blocks the release of glutamate, CGRP, and substance P [115,116,117,118]. Readers are directed to a recent review by Matak et al. [92] for details on the mechanism of action of BoNT in pain. 

A series of investigations by Burstein’s group highlighted that BoNTA acts on the C-fibers to reduce pain and that TRPV1 and TRPA1 channels play a critical role [119]. In addition, this group has proposed that BoNTA is capable of altering inflammatory gene expression and immune cells in migraine prevention, where it can reduce pre-existing inflammation [120].

Considering the evidence for the blockade of the vesicular release of substances by BoNTA presented above, it is not implausible to propose that BoNTA may block the release of substances that contribute to the sensation of itch [52]. To explain this, it is essential to present how itch is generated and transmitted. The section below presents what is known about pathways leading to an itching sensation. 

### 3.2. Itch Mechanisms

**Figure 2:**
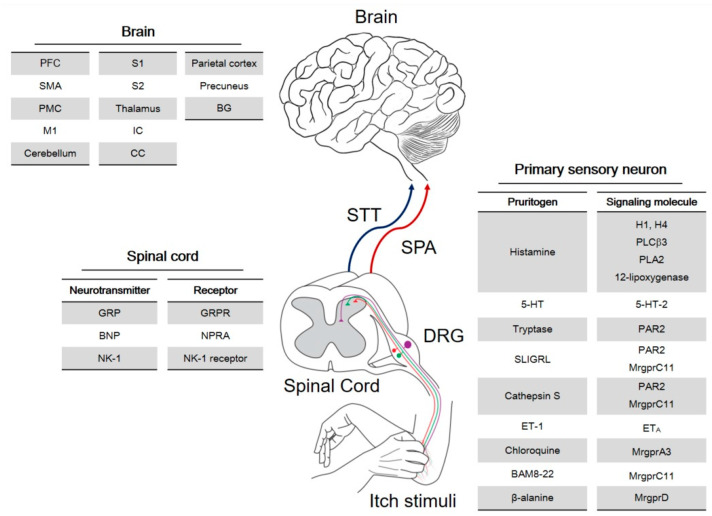
Itch signaling pathway: Schematic illustrating the transmission of itch from the primary sensory neurons to the brain. Itch stimuli (pruritogens) activate itch-sensing neurons in the dorsal root ganglion (DRG) that innervate the skin, which then stimulate second-order neurons in the spinal cord and multiple brain regions. Indicated in the tables are pruritogens, itch-selective molecules and receptors expressed in the primary sensory neurons and spinal cord, and brain regions activated by the cutaneous application of a pruritogen. STT, spinothalamic tract; SPA, spino-parabrachio-amygdaloid pathway; PFC, prefrontal cortex; SMA, supplementary motor area; PMC, premotor cortex; M1, primary motor cortex; S1, primary somatosensory cortex; S2, secondary somatosensory cortex; CC, cingulate cortex; IC, insular cortex; BG, basal ganglia; GRP, gastrin-releasing peptide; GRPR, gastrin-releasing peptide receptor; BNP, B-type natriuretic peptide; NPRA, natriuretic peptide receptor A; NK-1, neurokinin-1; H1, histamine H1 receptor; PLCβ3, phospholipase C β3; PLA2, phospholipase A2; 5-HT, 5-hydroxytryptamine (serotonin); 5-HT-2, 5-HT receptor subtype 2; PAR2, protease-activated receptor 2; Mrgpr, Mas-related G-protein-coupled receptor; ET-1, endothelin-1; ETA, endothelin-1 receptor A; BAM8-22, bovine adrenal medullary peptide 8–22. Reused from [125] under an open access terms of the Creative Commons Attribution Non-Commercial License, BMB Rep, Korean Society for Biochemistry and Molecular Biology.

Pruritus or itch is an uncomfortable sensation that generates a strong desire to scratch [121], which is seen in both humans and animals [122]. Itch is a unique sensory modality within the somatosensory system, and once it becomes chronic, it poses major distress and impairs the quality of life of the affected individuals [123]. In addition, people with chronic itch frequently suffer from self-harm when they are in the loop of uncontrollable itch–scratch cycles [124]. Itch is often associated with dermatological conditions, but it can also be a hallmark of systemic, neurological, and psychogenic conditions [123]. The mechanisms of itch have been extensively investigated in recent years, and as a result of the better understanding of itch pathways, several targeting sites and molecules have been identified and introduced to the field. Figure 2 presents a simplified sketch of the itch signaling pathway from the primary sensory neurons to the brain [125]. It is yet to be determined where and how exactly BoNT targets itch alongside this signaling pathway. Evidence is, however, being accumulated (Please refer to Section 4). 

**Figure 3:**
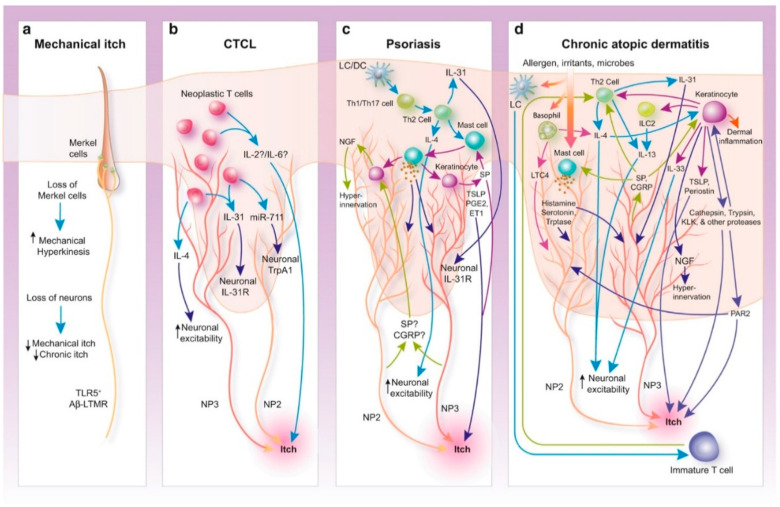
Mechanisms of chronic itch: (**a**) Age-related loss of touch-sensitive Merkel cells is associated with increased mechanical hyperkinesis. However, the loss of TLR5 Aβ-LTMRs that detect mechanical itch leads to the loss of mechanical itch sensitivity and the attenuation of chronic itch. (**b**) The current understanding of CTCL itch occurs through two main pathways. First, IL-31 released by neoplastic T cells can directly activate neuronal itch receptors, whereas IL-4, IL-2, and IL-6 enhance itch. (**c**) Psoriasis-associated itch mechanisms are not completely understood. Endogenous pruritogens, including IL-31, TSLP, and mast cell–derived PGE2 and ET1, have all been implicated, whereas NGF and type 2 cytokines may further potentiate itch by enhancing neuronal excitability. (**d**) AD-associated itch is primarily generated by type 2 cytokine-induced itch neuron excitability and reciprocal modulation by neurons. Endogenous pruritogens, released by immune cells and keratinocytes, include peptides, proteases, and mast cell-, basophil-, and ILC2-derived factors. AD, atopic dermatitis; CGRP, calcitonin gene-related peptide; CTCL, cutaneous T-cell lymphoma; DC, dendritic cell; ILC, innate lymphoid cell; KLK, kallikrein; LC, Langerhans cell; LTC4, leukotriene C4; NP, nonpeptidergic; SP, substance P; Th, T helper; TLR, Toll-like receptor. Illustration assistance provided by Ruvido Medical Illustration. Reused from [129] with permission (license number: 5379421293131) from Elsevier and Copyright Clearance Center.

Historically, two main categories of itch have been defined, histaminergic and nonhistaminergic itch [126], which are closely related but act independently from each other as two separate pathways. Chronic pruritus is proposed to involve the nonhistaminergic pathway [127]. In itch transmission, two families of receptors are found to contribute: G protein-coupled receptors (GPCRs) and transient receptor potential (TRP) channels [127,128]. Numerous molecules are found that activate these receptors, for example, at-the-periphery [129] histamine; serotonin; nerve growth factor; interleukins IL-4, 13, and 31, among many others. The itch sensation can be induced by the direct or indirect activation of these receptors and channels and activators of these are released from various cells, including T-cells, mast cells, and keratinocytes [129]. The number of players emphasizes that there is no singular cause of itch, and as a consequence, mechanisms underlying various chronic itch conditions differ [129]. Figure 3 depicts detailed peripheral mechanisms underlying some of the chronic itch conditions [129].

Several reviews are available, and readers are encouraged to look deeper into itch pathogenesis and treatments [123,125,129,130,131]. 

Antipruritic effects of BoNT have been presented in clinical cases in the literature (see Section 2). Investigators have tried to identify how BoNT can reduce or stop itch. To understand the mechanisms underlying the antipruritic effects of BoNT, cell-based studies, laboratory animal investigations and human experimental models have been employed to provide the mechanism-based evidence that is presented below.

## 4. Mechanisms Underlying the Antipruritic Effect of BoNTs

As presented above, merging known mechanisms underlying BoTN effects at the cellular–molecular level, and mechanisms of itch generation and transmission can help understand the mechanism(s) underlying the antipruritic effect of BoNT. This knowledge, combined with evidence of the antipruritic effects of BoNTs at the clinic is beneficial and important because it can potentially strengthen, accelerate, and facilitate the current efforts towards further investments in pushing the field forward for the potential approval of BoNTs for itchy conditions. In addition, it can advance the scientific field in terms of gaining a better understanding or providing evidence of neuronal, glial, and immune-modulatory systems involved in itch and targeting them with BoNTs and compounds similar to BoNTs. Further information, such as dosing, interval, and safety information, can also be gained through such preclinical studies. Experimental models of itch [132] are helpful in this regard, as clinical conditions of itch accompany several other confounding factors [133] that cannot be eliminated while studying itch mechanisms. The concept of itch models is somewhat similar to the concept of modeling other medical conditions, e.g., pain. In experimental human models of pain [134] and central sensitization [135], healthy volunteers act temporarily as subjects for the provocation of pain, which is an ethical, controlled, and short-term condition. In this scenario, the application of a chemical algogen or other types of stimuli (thermal, ischemic, mechanical, or electric) produces pain and other measurable outcomes, such as pain sensitivity, measured by a visual analog scale (VAS) and vasomotor responses. In these experimental models, responsiveness to various analgesics [136], including BoNT, has been evaluated [110,111,112,137].

### 4.1. Human Surrogate Models of Itch—Antipruritic Effects of BoNT

Human surrogate models of itch have provided a platform for studying new antipruritic compounds, as well as the investigation of the mechanisms underlying antipruritic effects of already approved compounds for other conditions, for instance, BoNTs. Experimental itch [132,138,139] in humans is induced over a short period and is usually assessed psychophysically. Itch has been provoked by the application of electrical [140], mechanical [141], and chemical stimuli, the latter being through the application of, for example, histamine [142,143], cowhage [144,145], capsaicin [146], BAM8-22 [147], β-alanine [148], and serotonin [149].

In 2009, we tested if the administration of BoNTA subcutaneously can reduce itch in a human model of itch [83]. The study recruited 14 healthy male subjects, and the itch was artificially provoked by histamine, which was delivered to the volar forearm skin using a prick test [83]. An amount of 5 U of BoNTA (BOTOX^®^, Allergan) or a similar volume of saline (control) was administrated prior to the histamine provocation test [83]. Baseline assessments were conducted, and itch intensity and neurogenic inflammation produced by the histamine prick test were evaluated one day, three days, and a week after the administration of BoNTA or saline. The results of this study [83] showed that BoNTA was capable of diminishing itch intensity and reducing the area of itch perception compared with saline at all time points post-treatment. The itch resolution time was also shorter in BoNTA-treated areas, and the maximum effect was seen on day 7. The flare area (observable skin reaction in the form of redness) was smaller on the BoNTA-treated arm at all post-treatment time points. Histamine-induced elevated blood flow and skin temperature subsided following the application of BoNTA, with the largest effect seen on days 3 and 7. This study showed that BoNTA can inhibit histaminergic itch in humans [83].

Later, in 2017, a group of researchers [150] applied a non-histaminergic model of itch in healthy humans and investigated if BoNTA can also exert antipruritic effects. In this study, 35 (16 males and 19 females) healthy volunteers were enrolled, and experimental itch was provoked by the application of cowhage [145] (*Mucuna pruriens*). Arthur and Shelley, in 1955 [151], were the first to discover that cowhage induces itching and scratching. This is the effect of mucunain, which is the active component of cowhage and is chemically classified as a cysteine protease. This substance binds to proteinase-activated receptors 2 and 4, so-called PAR-2 and PAR-4, respectively [134]. This model is proposed to mimic chronic itch conditions in humans, which are often non-histaminergic and do not respond to antihistamines [152]. In this study, 10 U of BoNTA (BOTOX^®^, Allergan Inc., Irvin, CA, USA) administered intradermally could reduce the itch intensity at all time points compared with the saline that was used as the control treatment. Sensory tests in this study [150] included skin temperature sensitivity, pain, and itch following cowhage and post-treatment, and measurements were performed at baseline, week 1, month 1, and month 3 post-treatment. This study [150] provided evidence for the long-term effect of BoNTA against itch, lasting 3 months following a single application.

Taken together, these two studies, using both histaminergic and non-histaminergic itch models in humans, showed the antipruritic effects of BoNTA, where it reduced itch and related symptoms (neurogenic flare, skin temperature, skin blood flow, and paresthesia in the form of hyperknesis and alloknesis) in human skin as early as 1 week, and up to 3 months in the cowhage model [150].

This field is still open for further investigation by applying these or other surrogate models of itch, combined with other subjective (psychophysics and quantitative sensory testing) and objective measures, for example, the bioanalysis of biomarkers through microdialysis, skin micro biopsy, or imaging studies. We employed the human dermal microdialysis technique [153] and presented that BoNTA inhibits the release of neurotransmitters, e.g., glutamate, in human skin [114]. It is valuable to understand how BoNTs interact with various cell types, including immune cells and nerve endings. The modeling of some dermatological conditions that accompany itch is not ethical in healthy humans; therefore, it is proposed that at least selected measurement techniques be used in patients pre- and post-BoNT treatments for the identification of the mechanism of action. These conditions include, but are not limited to, post-herpetic neuralgia, notalgia paresthetica, Hailey–Hailey disease, and psoriasis.

### 4.2. Rodent Surrogate Models of Itch—Antipruritic Effects of BoNT

Animal studies [154] are of great importance to understand the underlying mechanisms of pathogenesis and the action of drugs in humans. These models, however, can only mimic limited aspects of pathogenesis and often accompany translational challenges from animals to humans due to multiple factors, including species differences [155]. Considering these limitations, several itch models [132] have been developed and tested in different animals, mainly rodents. These models resemble itch of both an acute and chronic nature and have been employed to understand how itch is transmitted and how it can be targeted at various points of transmission [156]. Followed by the discovery of Arthur and Shelley [151] about the pruritogenic properties of cowhage, in 1963, Joglekar and colleagues [157] applied cowhage ointment (5%) topically and reported the provocation of itching and scratching in dogs. Since then, several models have been introduced, for example, bombesin-induced itch in rats [158] and intrathecal morphine injection in monkeys [159]. An easy subcutaneous injection of pruritogen in experimental mice was introduced in 1995 by Kuraishi and his colleagues [160], in which they selected the necks of mice where scratching bouts using the hind legs could be quantified as the outcome. This model and similar ones helped in the identification of neurotransmitters and neuromodulators of itch, as well as itch signaling pathways. A long list of substances has been used to induce itching and scratching behavior, for example, serotonin [161], chloroquine [162], SLIGRL (Ser–Leu–Ile–Gly–Arg–Leu, a proteinase-activated receptor-2 agonist) [163], interleukin-31 (IL-31) [47], and phoenixin [164].

In addition, it is possible to develop animal models of diseases that accompany itch and other symptoms to study mechanisms and treatment options. Among many disease models, NC/Nga mice are, for example, used for studying AD-like skin lesions and atopic dermatitis [165]. The modeling of psoriasis [166] has also been achieved with the aid of a Toll-like receptor 7 agonist, imiquimod, which is applied to the backs of mice. Another model is the dry skin model [167], which is induced by the application of a 1:1 mixture of acetone and ether to the nape of mice’s necks. In addition to in vivo models, and the behavioral outcomes of itch-evoked scratching [122], other measurements can be planned, such as in vitro investigations, to help in the identification of cellular and molecular aspects and changes following the application of various compounds to provoke or inhibit itch.

Although the antinociceptive effects of BoNTs have been extensively tested in animal models of pain [168,169,170], the antipruritic effects of BoNTs have only been investigated in a couple of itch provocation and disease model studies. These studies have proposed the potential antipruritic mechanisms of BoNTs to be the blockade of neurotransmitter release, the blockade of mast cell degranulation, the downregulation of TRPs, and the inhibition of neuroimmune key players, such as IL-17. In the following, these findings are presented in detail.

In the study by Ramachandran and colleagues [171], compound 48/80 or chloroquine was injected intradermally into mice to induce itch. The compound 48/80 is known to induce mast cell-dependent scratching, while chloroquine is known to be a mast cell-independent compound to provoke itching and scratching. The researchers applied both BoNTA_1_ and BoNTB_1_ (1.5 U, intradermal injection) to test their effects on days 2, 7, 14, and 21. In this study [171], saline was used as a control. They also investigated human and murine mast cells in culture and investigated the direct effect of BoNTs in vitro [171]. This study identified the interaction of BoNTs with mast cells, and findings demonstrated that both compound 48/80 and chloroquine provoked itching and scratching behavior, and BoNTs could reduce these outcomes. An explanation of the mechanisms underlying these effects, however, did not appear to be straightforward [171]. Pre-treatment with BoNTA_1_ and BoNTB_1_ inhibited compound 48/80-provoked mast cell degranulation in culture. This finding indicated that these toxins may directly affect mast cells and prevent the degranulation of these cells. Since BoNTs target SNAREs, the authors [171] investigated the target component of this complex by simulating the inhibition of SNAP-25 or VAMPs by BoNTs A_1_ and B_1_, respectively. The mRNA expression of SNAP-25 in mast cell cultures was very low in both cases of mice and human cell cultures. To confirm this finding, the authors [171] performed immune staining for SNAP-25 and VAMPs, and the cleavage of these isoforms became evident. The Western blot result, however, did not show SNAP-25 in mast cells, and there was no indication of BoNTB_1_ on mast-cell-expressed VAMPs. This finding [171] raised speculation that BoNTs may inhibit vesicular release from mast cells by a mechanism other than the SNARE-related mechanism. The authors [171] speculated that TRP channels might be involved [172,173], based on the literature exhibiting that BoNTA_1_ inhibits TRPV1 receptor function. According to the authors [171], BoNTs may also inhibit the depolarization-evoked calcium currents in mast cells. This can explain the inhibitory effect on the compound 48/80–evoked response, because compound 48/80 induces mast cell degranulation via calcium-dependent exocytosis [174,175]. Further investigation is required to determine if BoNTs inhibit the degranulation of mast cells in itch via direct or indirect mechanisms.

Another study [176] investigated the antipruritic effects of BoNTA in itch models of acute and chronic itch in mice. The authors applied compound 48/80, chloroquine, and a mixture of acetone–diethyl and ether–water to provoke itch [176]. Intradermal BoNTA could present a long-term inhibition of itch in both compound 48/80 and chloroquine models of acute itch. The effect was seen from day 1 to day 14. Itch induced by acetone–diethyl ether–water was also reduced by BoNTA pretreatment up to day 14. To study the potential mechanisms, the authors [176] looked at the levels of receptor expression in mice dorsal root ganglia, where they found that BoNTA could reduce the expression of TRPV1 and TRPA1 in both acute itch models provoked by compound 48/80 and chloroquine. However, in the dry skin model of chronic itch, BoNTA only reduced the DRG upregulation of TRPA1 [176]. Collectively, based on these findings [176], the authors proposed that, at least in part, the downregulation of TRPA1 and TRPV1 in DRG can contribute to the antipruritic effects of BoNTA.

A recent study [177] employed one of the disease models, psoriasis, and investigated if BoNTB could interfere with the immune axis of IL-23/Th17, which is proposed to act as one of the primary modulators in psoriasis [178]. The study investigators [177] applied imiquimod to artificially model psoriasis-like dermatitis [179] in mice. Following pre-treatment with BoNTB, they found the significant suppression of cytokine production in skin lesions. In addition, the cell counts for CD4+ T cells, CD11c+ dendritic cells, and IL-17 were reduced dramatically [177]. The authors also found that BoNTB reduced the expression of substances P and CGRP on PGP9.5+ nerve fibers, which are reported to be increased in the lesions of psoriatic skin [180,181]. Results from this study [177] emphasize the importance of the neuroimmune system in psoriasis and that BoNTB via inhibitory action on this system could reverse the condition of lesioned skin. In this study, however, itch or scratch behavior was not measured, and the effect of BoNTB was mainly based on the scoring of erythema, scale, the thickness of skin, and overall scale [177]. Therefore, it is speculated that BoNTB in this study might have also reduced itch and scratch behavior in mice.

The cleaving effect of BoNTs on SNARE has also been proposed as an ideal long-lasting solution to break a vicious cycle of immune–nerve communication in pathologic conditions such as AD [182]. BoNTA also blocks substance P and CGRP release [183], similar to what is observed for BoNTB [177]. BoNTA can also prevent the upregulation of TRPA1 and TRPV1, which occurs following the activation of Th_2_ by cytokines [184] and breaks the vicious cycle of neuro-immune contribution in AD [182]. It is reasonable to consider that when SNAP-25 is cleaved by BoNTA, the release of natriuretic peptide (BNP) from pruriceptive neurons is inhibited [185], and this results in blocking itch transmission. BNP is known to potentiate TRPV3 expression on keratinocytes, and it is proposed that BoNTs, most likely B and D serotypes, can prevent this by cleaving VAMPs [186] (vesicle-associated membrane protein).

Although a large amount of research work remains to be done regarding the antipruritic effects of BoNTs, we can benefit from the knowledge accumulated about antinociceptive effects of BoNTs at the cellular and molecular levels. Considering this, one study looked into the neuron–glia modulation of LPS-induced pain and the effect of BoNT [187]. Results from this study showed that BoNTA reduced the LPS-induced phosphorylation of p38, ERK1/2, and NF-B and blocked the release of pro-inflammatory IL-1, IL-18, and IL-6. Interestingly, it also blocked the release of anti-inflammatory IL-10 in microglia [187]. The authors [187] explained the action of BoNTA in glial cells to be related to the activation of TLR2 and TLR4 receptors. An interesting finding of this study [187] was that the activation of TLR2 in astroglia requires a microglial TLR4 receptor [187]. This can be similarly investigated in itch provocation models to further identify glial roles in itch [188] and the modulatory role of BoNTs on these cells in the peripheral and central nervous system to inhibit itch. For example, we investigated the expression of SNAPs on satellite glial cells (SGCs) in the trigeminal ganglia and found that BoNTA can inhibit the vesicular release of substances from these cells in vitro [113]. Such findings can add value to the identification of peripheral components of itch transmission in sensory ganglia (trigeminal and dorsal root ganglia). Moreover, differences and commonalities can be investigated to determine if the region plays a role in anti-pruritic response within spinal-innervated or trigeminal-innervated areas. This type of investigation can, for example, also address potential mechanisms underlying conditions such as psoriasis, where inverse psoriasis [189] might show a better response to BoNTs compared with psoriasis.

**Figure 4:**
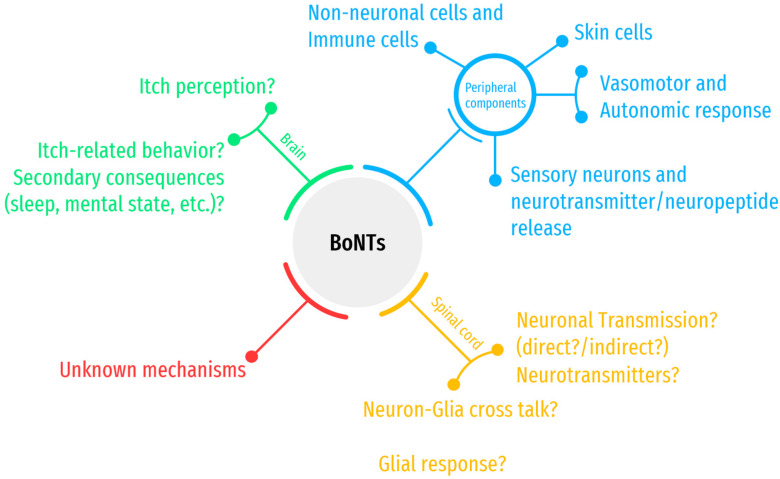
Proposed antipruritic mechanisms of BoNTs. Peripheral components of itch have been studied extensively, and the literature provides evidence of the beneficial effects of BoNTs on these components, such as blocking the release of neurotransmitters and neuropeptides (pruritogen, itch mediator) and blocking the vasomotor and autonomic components of itch. Spinal cord and brain components of itch are being actively investigated, and animal experiments and imaging studies are revealing components of itch mechanisms, transmission, and perception in addition to itch behaviors and emotional response. Limited evidence in the literature points to potential direct/indirect itch inhibitory effects of BoNTs in the central nervous system and these elements (transmission, perception). Some unknown complex mechanisms might also exist that can indirectly lead to antipruritic effects of BoNTs, which are yet to be determined. For details of this simplified schematic, please refer to the text.

The central effects of BoNTs, following their peripheral administration, have been the subject of debate in the literature, and a consensus has not yet been reached as to their direct or indirect effects [190,191,192]. A 2018 review by Caleo and Restani [191] summarizes studies that have so far provided evidence of the retrograde transport of BoNTA after its peripheral injection and that this process might contribute to the clinical effects of BoNTA through a direct action on the central circuits [191]. It is yet to be determined if this or other mechanisms can be involved in the effects of centrally mediated itch in clinical conditions such as chronic neuropathic itch [57,193]. Independent of the direct or indirect mechanism of action, it has been proposed that central alterations made by BoNTs can offer therapeutic applications to pathological conditions that are maintained by maladaptive plastic changes, such as neuropathic pain [194,195] or neuropathic itch [57,193], which are both difficult to treat. The accumulation of mechanism-based evidence with high-quality data can potentially lead to the approval of BoNTs for the treatment of itch [43,60,67] most likely in chronic itch states and in troublesome conditions that are resistant to other treatment options. Figure 4 depicts an overview of the potential action points of BoNTs to exert antipruritic effects. Questions marks indicate uncertainty or unknown areas in the available literature, where further investigation is required for clarification.

## 5. Conclusions and Future Perspectives

Most of the clinical studies presented in the literature show potential for the beneficial effects of BoNTs against itch and dermatological pathologies with itch as a cardinal symptom. High-level evidence, resulting from well-designed conducted studies, will most likely lead to the enhancement of the grade of recommendation to the point that BoNTs can be approved for their antipruritic efficacy [60,67]. The long-lasting effects of BoNTs after administration appear to be in favor of patient compliance, but the cost might be inhibitory for regular use, in addition to responsiveness to other available compounds with comparative effects to BoNTs. Theoretically, it makes sense to evaluate each pruritus condition prior to the selection of or switching to BoNTs. Safety considerations are also crucial. The overall consensus is that BoNTs are generally safe if used following the recommended dose and interval, but the risks and benefits must be weighted and justified in each case [196]. It is predicted that the present and near-future focus will be directed towards conditions with itch where stronger evidence is available for the choice of BoNTs against itch. This is a positive reason to conduct studies with a proper design. Points to be considered can be moving toward study designs that can provide higher-grade evidence, i.e., randomized controlled trials (RCTs) instead of observational, open-label, or case-based studies. Blinding, and considering a placebo or control arm would minimize bias and enhance the validity of results. This method can also help in determining the existence or proportion of a placebo effect. Power calculation, the effect size for treatment, and sample size determination will result in sufficient study size and limit the potential for false positive or false negative results as a consequence of poor study power or small sample size. A well-defined inclusion and exclusion criteria will also help in recruiting study participants who are the target group for testing the study hypothesis as if BoNTs can exert beneficial antipruritic effects. In this line, multi-center studies that enable the recruitment of a large number of participants can be helpful. Confounding factors, such as gender, age, and ethnic background of participants must be considered and reported to permit the identification of responders versus non-responders. The administration dose and interval of dosing (single or repeated injections), and optimal way of delivery (e.g., intradermal or subcutaneous), must also be justified and included in the study design. Current studies lack a unified description of the location and dosage for the application of BoNTs. In terms of outcome measures, the antipruritic effect seems to be a proper and important primary outcome. However, discrepancies still exist in the application of validated or approved measurement tools. Hence, it is valuable to describe in sufficient detail how the outcomes are measured. Secondary outcomes can also be defined and described. For example, sleep quality can be determined as a secondary outcome for the antipruritic effect as itch disturbs sleep and reduce the quality of sleep. Well-defined study outcomes would allow for comparisons between studies or meta-research (e.g., meta-analysis) in the future. BoNTs injections are considered safe and well-established in the cosmetic field for example for facial wrinkles, however, it is important to consider safety measures in the next trials investigating the antipruritic effects of BoNTs. Collecting information such as patient satisfaction, or the presence of tingling or bleeding during the injections can help in the determination of benefits versus challenges or any potential risks.

The clinical practice reports show the main use of BoNTs for localized chronic pruritus in a diverse range of dermatological conditions such as burns, scars, lichen simplex, and inverse psoriasis. However, the therapeutic response to BoNTs for itch of non-dermatological origin [197] and itch as a systemic disease [198] (e.g., renal, liver, endocrine–metabolic diseases, and hematologic–lymphoproliferative diseases) can also be investigated. BoNTs could be a potential option when multiple symptoms are presented at the same time in patients, for example, in cosmetic and non-cosmetic surgeries, where multiple complex mechanisms including sensory alterations are involved (e.g., wound healing and ulcer treatment [199]. It has even been claimed that “*if there was ever a drug that was likely to affect every cell of the body, this is BoNT*” [200].

Preclinical surrogate models of itch in humans and laboratory animals confirm that BoNTs are capable of preventing itch. Animal studies, in particular, could enrich our understanding of the mechanisms of BoNTs antipruritic effects, and provide evidence on acute and chronic itch prevention using BoNTs. These studies have also provided a platform for the investigation of the peripheral and central effects of BoNTs and their potential antipruritic mechanisms. The current understanding is that BoNTs potentially inhibit itch at several levels, through the blockade of neurotransmitter and neuropeptide release; the blockade of mast cell degranulation; the downregulation of TRPs; and the inhibition of key players in the neuro-immune system, such as cytokines and interleukins involved in itch, as well as the Th_2_ cytokine-induced release of itch-promoting substances, such as BNP. Independent of uncertainty around the direct or indirect central role of BoNTs, accumulating evidence highlights the potential role of peripheral and central glia in itch and the potential modulatory role of BoNTs.

Surrogate and preclinical models of itch can help in further studying the roles of neuronal and non-neuronal (e.g., glia) components and their interactions in the development and maintenance of chronic itch, and how BoNTs and other potential targets can prevent or stop it. The field of itch pathogenesis and treatment [130] is active, and new formulations of botulinum toxins with desirable safety profiles and enhanced potency are emerging [201]. The crossover of these two fields offers an exciting horizon for multifold potentials in the future of chronic itch treatment and widening the medical indications of BoNTs.

## Figures and Tables

**Table 1:** Summary of studies examining the effect of botulinum toxin type A in chronic pruritus. CR, case report; POS, prospective observational study. **∗** Determined based on the Oxford Centre for Evidence-based Medicine levels of evidence. Reused with permission (license number: 5376990524166) from Elsevier and Copyright Clearance Center.

Grade of Recommendation ∗	Study Design	Diagnosis	N	Regimen	Degree of Pruritus Reduction	Reference
**C**	POS	Lichen simplex	4	100 U/mL BoNT/A spaced 2 cm apart	One treatment led to complete relief of symptoms lasting 4 months	[58]
**C**	POS	Partial-thickness to full-thickness burns	9	25 U/mL BoNT/A (spacing unknown)	One treatment led to a reduction in symptoms to <3/10 lasting an average of 6.3 months	[68]
**C**	POS	Inverse psoriasis	15	20 U/mL BoNT/A spaced 2.8 cm apart	One treatment led to a reduction in the mean visual analog score to 2.1/10 lasting 3 months	[41]
**C**	POS	Notalgia paresthetica	5	40 U/mL BoNT/A spaced 2 cm apart	In 3 patients: 1 treatment led to “symptomatic improvement” lasting 1 month; in 2 patients: 1 treatment led to “worsening of pruritus”	[69]
**C**	POS	Notalgia paresthetica, meralgia paresthetica, and neuropathic itch	6	0.27–0.47 U/mL BoTN/A (spacing unknown)	One treatment led to a 28% reduction in the mean visual analog score lasting ≥6 weeks	[70]
**C**	POS	Notalgia paresthetica	2	1.3 U/mL BoNT/A spaced 2 cm apart	First patient: 1 treatment led to complete relief of symptoms lasting ≥18 months; second patient: 2 treatments spaced 18 months apart led to complete relief of symptoms lasting an unknown duration	[42]
**D**	CR	Brachioradial pruritus	1	33.3 U/mL BoNT/A spaced 1.5 cm apart	One treatment led to complete relief of symptoms lasting 6 months	[71]
**D**	CR	Intractable pruritus after facial surgery	1	15 U BoNT/A (unknown dilution or spacing)	One treatment led to a “significant reduction” in symptoms lasting 2 months	[72]
**D**	CR	Axillary granular parakeratosis	1	50 U BoNT/A (unknown dilution or spacing)	One treatment led to a complete relief of symptoms lasting 4 months	[73]
**D**	CR	Inverse Psoriasis	1	100 U BoNT/A (unknown dilution or spacing)	One treatment led to a complete relief of symptoms lasting ≥1 month	[74]
**D**	CR	Hailey–Hailey disease	1	200 U BoNT/A (unknown dilution or spacing)	One treatment led to the complete relief of symptoms lasting 3 months	[75]

## Data Availability

A part of this work was presented during the invited plenary lecture, at the 6th International Conference of Basic Science and Clinical aspects of Botulinum and Other Neurotoxins, Toxins 2022, organized by the International Neurotoxin Association in New Orleans, Louisiana, US (27–30 July 2022).
